# Supplementary material for: Quality of life and associated factors among the youth with substance use in Northwest Ethiopia: Using structural equation modeling
Source: PLoS One. 2022 Sep 20;17(9):e0274768. doi: 10.1371/journal.pone.0274768 (PMC9488770; doi:10.1371/journal.pone.0274768)
Supplement: Attachment — Submitted filename: Author respons.docx [file pone.0274768.s002.docx]

**Author Response Letter
Manuscript ID: PONE-D-22-02286**
**Title: Quality of life and associated factors among substances user youths in Northwest Ethiopia: Using Structural Equation Modeling**

Dear editor(s) and reviewers
First for all the authors would like to thank the editor(s) and reviewers for your precious time, thoughtful comments and constructive suggestions, which help to improve the quality of this manuscript. The corresponding changes and refinements made in the revised manuscript are summarized in our response below.

**Q:** reviewer’s comments, suggestions and questions

**Response**: authors response based on the editors/ reviewer questions and comments
Journal Requirements
Q1. Please ensure that your manuscript meets PLOS ONE's style requirements, including those for file naming. The PLOS ONE style templates can be found at
Response: To address the journal requirements, correction and revision have been made in the revised document.

Q2: Please include additional information regarding the survey or questionnaire used in the study and ensure that you have provided sufficient details that others could replicate the analyses. For instance, if you developed a questionnaire as part of this study and it is not under a copyright more restrictive than CC-BY, please include a copy, in both the original language and English, as Supporting Information.

**Response:** Thank you for the comments, we have revised the manuscript accordingly. For example, the survey questionnaires for this study consists of five components such as: socio-demographic characteristics, family-related questions, social support, psychotic symptoms, and the WHO-QOL tool questions. The sociodemographic and family related questions were developed from the previous literatures. Whereas the standard questionaries were used for the measurements of social support, psychotic symptoms, and health related quality of life.

We have submitted the survey questionary we used for this study as supplementary file.

Q3. Thank you for stating the following financial disclosure:
**Response:** we update the financial disclosure in the revised manuscript and cover letter.

Q4. In your Data Availability statement, you have not specified where the minimal data set underlying the results described in your manuscript can be found.

**Response:** in the revised manuscript we updated the Data Availability statement as “The datasets supporting the conclusions of this manuscript are available upon request to the corresponding author”.

**Reviewers' comments**
**Reviewer #1 Comments and Authors Response**

Q1: please improve result section with apply and mention statistical.

**Response:** Thank you so much for your important comment. We have already revised the result in abstract section in the revised manuscript.

**Q2: How do they reach the conclusions in the abstract?**

**Response:** Based the study results and by considering the gap, we tried to conclude our findings.

**Q3**: please use update references in this section.

**Response**: Thank you so much for your important comment and for giving us the opportunity to improve the revised manuscript. We have revised the background section with updated reference based on your recommendation.

**Q4**: what are your hypotheses? please mentioned its in this section or methods section.

**Response**: Thank you again for asking these questions and giving us an opportunity to improve the revised manuscript. We have added the hypotheses of the study in the revised manuscript.

The study hypothesis was:

1) Null hypothesis = Substances user youths have good health-related quality of life

Alternative hypothesis = Substances user youths have poor health-related quality of life

2) Alternative hypothesis = Health related quality of life is associated with socio-demographic, family related, and psychosocial factors

**Q5: what’s are response rate in your study?**

**Response**: since our minimum sample size to run the SEM model was 370, we have invited 380 participants for our study. Three hundred seventy-two (372) youths were willing and interviewed the questionnaires with the response rate of 97.8%.

**Q6**: sampling technique and study setting very vague. please explain more. Geographical cluster of each district, is setting of your study, or special centers of "Kebele" are your setting?

**Response**: sorry for this vague information. The special centers of Kebele are our study settings. The study employed multi-stage sampling technique. We stand from central Gondar zone and selected 6 districts, then number of Kebeles are also selected from these districts. There are also special centers “Ketenas” inside Kebele. Here we have recruited our participants from the Ketena’s in the form of cluster by considering a proportional allocated sample in each Kebeles.

**Q7**: Were these questioner adapted from an existing tested questionnaire or did the authors develop it themselves, tested it?

**Response**: Thank you again for asking these questions. The tools used in this study were Standardized and validated previously except the socio-demographic and some personal related variables. But the internal reliability of the tools was tested in our setting.

**Q8**: Discussion does not explain the findings, Authors state other studies, but do not offer an explanation as to why they see the results that they see the results that they see

**Response**: Thank you again and for allowing us to improve the quality of the manuscript. We have already revised the discussion and updated the revised manuscript. Thanks again.

**Q9**: Please try to include more relevant literature

**Response**: Thank you again and for allowing us to improve the quality of the manuscript. We have added more literatures in the revised manuscript.

**Reviewer #2 Comments and Authors Response**

**Q1**: The study sample size is reasonable with adequate rule of thumb for 370 subjects. The model appears to have reasonable goodness of fit and RMSEA values. The analysis software used, which is routine in this context, appeared to adequately identify factors associated with health-related quality of life which is a major factor, naturally. Structural equation modeling was used to estimate the relationships among exogenous, mediating, and endogenous variables which is notably the purpose of such software. There are many variables and the presentation is a descriptive summary of the relationships sought by the authors. One can get lost in the presentation with so much being described.

**Response**: Many thanks for the thoughtful comments and constructive suggestions, which help to improve the quality of this manuscript.

**Q2**: The characterization of the strength of the interdomain correlations is troubling. The authors note for example that Inter-domain correlation showed that there was a statistically significant correlation between domains, there is a highly positive correlation between environmental health domain and psychological health domain (r=0.61, p<0.001) and as compared with other domain's, psychological health domain and social relation health domain had a relatively
weak correlation between them (r= 0.49, p<0.001). How are weak and strong being decided?

**Response**: The value of correlation coefficient (r) is lies between -1 and 1 inclusively. The Interpretation of r is as follow, perfect positive linear relationship (if r= 1) b/n the variables, no linear relationship (if r = 0), perfect negative linear relationship (if r = -1). Many scholars are agreeing to classify any value between correlation coefficient into strong positive (1 to 0.5), weak positive (0.49 to 0.1), strong negative (-0.5 to -1) and weak negative (-0.1 to 0.49).

**Q3**: The format of Table 3 makes it difficult to read and interpret the table. Please correct the alignment. There are typos in the manuscript. For example, in the title of Fig. 3, Self Reted should obviously be Self Rated. Parcel in Figure 4 is in the footnote, but it looks like Parcil in the figure.

**Response:** We thank the reviewer for the comment, we have gotten our mistakes, and we have corrected the comments in the manuscript. Thanks again.

**Q4**: The English should also be edited in places for clarity
**Response:** we are grateful for this comment which was our gap as it points to important issue to improve the readability of the text. After your suggestion, we have revised again the document, and then we have re-edited the grammatical error sentences by consuming more time and energy. We have also provided the document for professional language copy editor for advanced language edition. Based on this we have rewritten the whole document in more understandable to resolve language problems. Hence grammatical problems are resolved and changes/ modifications are highlighted by red color in the track changed file.

Furthermore, we have modified, added, and changed a lot things from the topic to references based on editor and reviewers’ suggestions. Finally, we thank the editor and reviewers for your kind comments, constructive criticism and useful suggestions for which we have used to improve our manuscript.
